# Imported Case of Poliomyelitis, Melbourne, Australia, 2007

**DOI:** 10.3201/eid1501.080791

**Published:** 2009-01

**Authors:** Andrew J. Stewardson, Jason A. Roberts, Carolyn L. Beckett, Hayden T. Prime, Poh-Sien Loh, Bruce R. Thorley, John R. Daffy

**Affiliations:** Eastern Health, Melbourne, Victoria, Australia (A.J. Stewardson, C.L. Beckett, P.-S. Loh, J.R. Daffy); Victorian Infectious Diseases Reference Laboratory, Melbourne (J.A. Roberts, B.R. Thorley); MIA Victoria, Melbourne (H.T. Prime)

**Keywords:** Poliomyelitis, human poliovirus 1, magnetic resonance imaging, polymerase chain reaction

## Abstract

Wild poliovirus–associated paralytic poliomyelitis has not been reported in Australia since 1977. We report type 1 wild poliovirus infection in a man who had traveled from Pakistan to Australia in 2007. Poliomyelitis should be considered for patients with acute flaccid paralysis or unexplained fever who have been to poliomyelitis-endemic countries.

The World Health Organization declared the Western Pacific region, including Australia, polio free in 2000 (*1*). The last known case of wild poliomyelitis in Australia occurred in 1977 (*2*). The National Polio Reference Laboratory (NPRL), in collaboration with the Australian Paediatric Surveillance Unit, coordinates surveillance for acute flaccid paralysis in children <15 years of age and investigates all suspected cases of polio (*3*). However, with increasing time since widespread poliomyelitis occurred in Australia, concern has been raised about whether poliomyelitis would be recognized (*4*). Indeed, we report a Pakistani patient in whom poliomyelitis was first considered only after magnetic resonance imaging (MRI).

## The Study

The patient was a 22-year-old Pakistani student in Melbourne, Australia; he had received at least 3 doses of oral polio vaccine as a child. On March 13, 2007, the student returned home to Kharian, Pakistan, to recuperate from herpes zoster, which had developed in February 2007. From June 4 through June 7, he toured the North West Frontier Province of Pakistan, then returned to Kharian. On June 22, he felt unusually hot. Over the next 2 days he had fever, diaphoresis, nausea, vomiting, and pain in his low back and legs. On June 24, he noted lower limb weakness, especially in his left leg. He was not hospitalized, and after several days, all symptoms except the pain resolved. On July 2, he traveled back to Melbourne.

Starting on July 3, the patient’s pain increased and lower limb weakness subsequently returned, accompanied by upper limb tremors. He had neither systemic symptoms nor bladder or bowel dysfunction. He was referred to Boxhill Hospital, Eastern Health, emergency department on July 6. Physical examination found that the patient’s legs were tender to palpation and that strength was mildly reduced in the entire left leg and proximal right leg. Lower limb reflexes and sensation, as well as cranial nerve and upper limb function, were within normal limits. Laboratory results of blood tests were within reference ranges.

**Figure 1:**
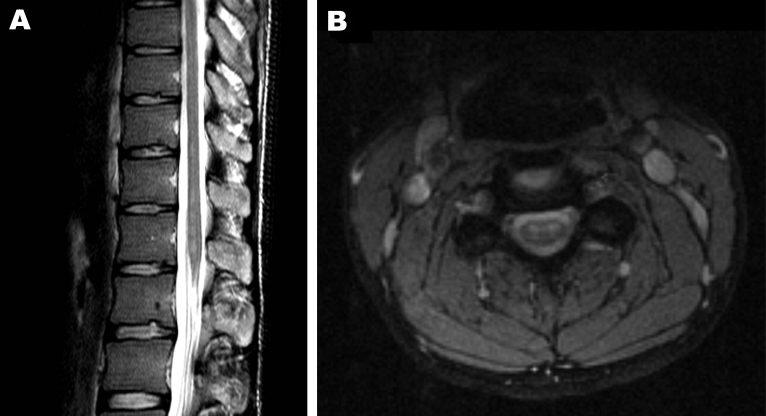
A) Sagittal image of the conus and B) coronal image of the cervical cord, demonstrating increased signal on T2 weighted sequences in the region of the anterior horns. There was no enhancement with contrast.

The next day, MRI was highly suggestive of poliomyelitis. It showed a globular pattern of increased signal on T2-weighted sequences, limited to the anterior horn region throughout the spinal cord, without enhancement with gadolinium (Figure 1). The Department of Human Services, Victoria, was notified of the clinical diagnosis of poliomyelitis; the patient was admitted to a single room, and contact precautions were instituted. Panenterovirus reverse transcription–PCR (RT-PCR) of feces, serum, and throat swab produced negative results, but the samples were forwarded to the NPRL for cell culture 2 days later (Table). Cerebrospinal fluid contained 1 × 10^6^ polymorphonuclear cells, 8 × 10^6^ lymphocytes, 24 × 10^6^ erythrocytes, a protein concentration of 1.9 g/L, and glucose levels within normal limits.

**Table:** Virus diagnostic tests for poliovirus conducted for 22-year-old male Pakistani student in Melbourne, Australia, 2007*

Specimen	Time after symptom onset, d	EV RT-PCR	Cell culture	PV1 ELISA	ITD PCR
Feces	15	Negative	Positive	PV1NSL	PV1 wild
Swab (throat)	15	Negative	Negative		
CSF	15	Negative	Negative		
Feces	17	Positive	Positive	Not tested	PV1 wild
Feces	25	Negative	Negative		
Feces	28	Negative	Negative		
Feces	30	Negative	Negative		
Feces	37	Negative	Negative		

*EV RT-PCR, panenterovirus reverse transcription–PCR for direct detection from original clinical specimen; PV1, poliovirus type 1; ITD PCR, intratypic differentiation PCR; PV1NSL, poliovirus type 1 non–Sabin-like; CSF, cerebrospinal fluid.

Given the patient’s recent history of herpes zoster, intravenous acyclovir (10 mg/kg every 8 hours) was begun to treat possible varicella zoster virus–related myelitis. The patient had recovered completely by 48 hours, and this treatment was stopped. Laboratory identification of wild poliovirus type 1 was reported on day 7 after hospital admission, 5 days after fecal samples were submitted. The patient was quarantined in the hospital until 2 fecal specimens taken 7 days apart were negative for poliovirus by cell culture and RT-PCR, a total of 34 days (Table). The public health response, directed by the Department of Human Services, included vaccination of potentially susceptible persons who had been exposed to the index case-patient and home quarantine of his household contacts until poliovirus shedding was excluded. No secondary cases were identified.

At the NPRL, the initial fecal specimen was extracted according to recommended procedures (*5*) and incubated with 4 continuous mammalian cell lines: L20B, RD-A, Hep2 Cincinnati, and human embryonic lung. After 4 days, enterovirus cytopathic effect was observed in all cell lines except RD-A. This finding was after passage onto fresh cell lines to reduce toxicity.

**Figure 2:**
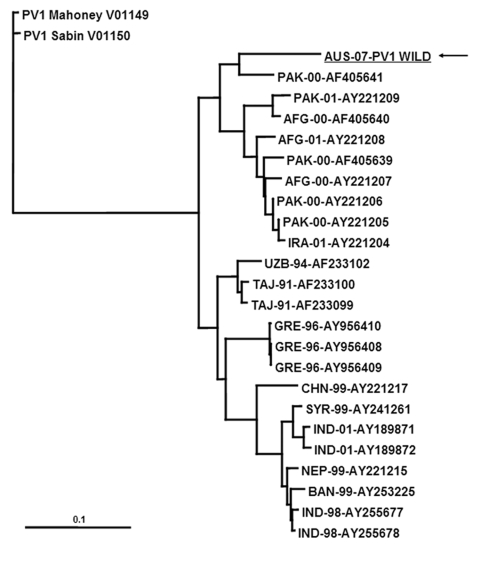
VP1 phylogenetic tree constructed by using cognate sequence available in the public domain was created by using the PHYLIP DNA maximum-likelihood algorithm with 100 bootstrap replicates. Marker represents relative phylogenetic distance. AFG, Afghanistan; AUS, Australia; BAN, Bangladesh; CHN, China; GRE, Greece; IND, India; IRA, Iran; NEP, Nepal; PAK, Pakistan; SYR, Syria; TAJ, Tajikistan; UZB, Uzbekistan. Scale bar represents number of nucleotide substitutions per site.

Confirmatory testing for enterovirus isolation and intratypic differentiation for poliovirus was performed (*5*). The virus was identified by RT-PCR and ELISA as non-Sabin–like poliovirus type 1. Sequences for comparative analysis of the virus capsid protein 1 genomic region were acquired by using the BLAST algorithm (www.ncbi.nlm.nih.gov/blast/Blast.cgi). Phylogenetic analysis showed a common ancestor with a wild poliovirus isolated from Pakistan in 2000 and clustering within a group of isolates originating in Pakistan, Afghanistan, and Iran (Figure 2). The National Polio Laboratory of Pakistan reported that the isolate from Australia had 98.8% nucleotide identity with isolates from Pakistan in 2006, which are not available in the public domain (S. Zaidi and S. Sharif, pers. comm.).

Acute- and convalescent-phase serum specimens were collected on days 15 and 47 after symptom onset, respectively, and tested for total immunoglobulin poliovirus neutralizing antibodies according to recommended methods (*6*). Antibodies to all 3 poliovirus serotypes were detected in both specimens. The potency of antibodies to authenticated Sabin poliovirus type 1 was 212 and 424 IU for the acute- and convalescent-phase samples, respectively. To have determined the titer of neutralizing antibodies at the onset of symptoms, earlier collection of acute-phase serum would have been needed.

Doubt was cast on the diagnosis of poliomyelitis because of the patient’s age, vaccination history, and the initial panenterovirus PCR–negative fecal specimen. However, previous vaccination does not exclude poliomyelitis; reduced seroconversion to polio vaccine is well described in polio-endemic regions (*7*). Furthermore, although this patient had received oral polio vaccine during childhood, he received no booster dose before his recent travel.

Several factors support an argument against the negative predictive value of the initial fecal panenterovirus RT-PCR. Fecal shedding of poliovirus by infected persons can be intermittent; hence, 2 fecal specimens from suspected case-patients are recommended (*5*). Panenterovirus RT-PCR was subsequently positive for the second fecal specimen, provided 2 days after the first. In retrospect, the virus titer in the first specimen may have been below the limits of detection for RT-PCR; this limitation was overcome by virus multiplication in cell culture. The panenterovirus RT-PCR is a heminested assay adapted from Zoll et al. (*8*) by addition of an internal second round primer. This assay’s performance in an external proficiency testing program conducted by the Royal College of Pathologists of Australasia (*9*) has met a high standard. Repeat testing of the first fecal specimen by this assay showed 1 of 15 replicates to be positive, consistent with a low poliovirus copy number.

Further, childhood vaccination may have attenuated the duration of virus shedding. Because the travel history, clinical illness, and radiographic appearance were highly suggestive of poliomyelitis, the presumptive diagnosis remained poliomyelitis until confirmation by fecal culture.

## Conclusions

Until polio is completely eradicated, polio-free regions remain at risk for importation and subsequent transmission of poliovirus. Poliomyelitis should be considered in patients who have acute flaccid paralysis or a febrile illness without an alternate diagnosis and who have travelled from countries with endemic poliovirus transmission; neither previous vaccination nor a single negative fecal enteroviral PCR excludes poliomyelitis. The high level of vaccination in Australia reduces the risk for local transmission after poliovirus importation. However, Australia has a large migrant population who might not be immune, and Aboriginal and Torres Strait Islanders exhibit reduced seroconversion rates after polio vaccination (*10*). Risk for local outbreaks can be minimized by widespread vaccination, early recognition of an index case, and prompt and appropriate public health measures.

## References

[1] Certification of poliomyelitis eradication. WHO Western Pacific Region, October 2000. Wkly Epidemiol Rec. 2000;75:399–400. 11189701

[2] Kennett ML, Brussen KA, Wood DJ, van der Avoort HG, Ras A, Kelly HA. Australia’s last reported case of poliovirus infection. Commun Dis Intell. 1999;23:77–9. 10323040 10.33321/cdi.1999.23.9

[3] Roberts J, Brussen KA, Ibrahim A, Thorley B. Annual report of the Australian national poliovirus reference laboratory, 2006. Commun Dis Intell. 2007;31:263–9. 17974218

[4] Durrheim DN, Massey P, Kelly H. Re-emerging poliomyelitis—is Australia’s surveillance adequate? Commun Dis Intell. 2006;30:275–7. 17120482 10.33321/cdi.2006.30.25

[5] World Health Organization. Polio laboratory manual, 4th ed. Geneva, Switzerland: The Organization; 2003 [cited 2007 Dec 14]. Available from http://www.who.int/vaccines/en/poliolab/webhelp/whnjs.htm

[6] World Health Organization. Polio laboratory manual, 2nd ed. Geneva, Switzerland: The Organization; 1997. WHO/EPI/GEN/97.01.

[7] Pallansch MA, Sandhu HS. The eradication of polio—progress and challenges. N Engl J Med. 2006;355:2508–11. 10.1056/NEJMp068200 17167133

[8] Zoll GJ, Melchers WJ, Kopecka H, Jambroes G, van der Poel HJ, Galama JM. General primer-mediated polymerase chain reaction for detection of enteroviruses: application for diagnostic routine and persistent infections. J Clin Microbiol. 1992;30:160–5. 1370845 10.1128/jcm.30.1.160-165.1992 PMC265013

[9] Royal College of Pathologists of Australasia. RCPA quality assurance programs PTY Ltd [cited 2008 18 Nov]. Available from http://www.rcpaqap.com.au/uploadedfiles/226_InformationBooklet.pdf

[10] Hanna JN, Sexton WL, Faoagali JL, Buda PJ, Kennett ML, Brussen KA. Immunity to hepatitis B, poliomyelitis and measles in fully vaccinated Aboriginal and Torres Strait Island children. J Paediatr Child Health. 1995;31:345–9. 10.1111/j.1440-1754.1995.tb00825.x 7576896

